# A Fusion of Taq DNA Polymerase with the CL7 Protein from *Escherichia coli* Remarkably Improves DNA Amplification

**DOI:** 10.3390/molecules29051145

**Published:** 2024-03-04

**Authors:** Zhongchen Li, Yaping Wang, Xiangyi Wang, Shuhui Niu, Zhenlong Su, Fei Wang, Jing Ni, Yan Gong, Ben Rao

**Affiliations:** 1 State Key Laboratory of Biocatalysis and Enzyme, Engineering Hubei Collaborative Innovation Center for Green Transformation of Bio-Resources, Hubei Key Laboratory of Industrial Biotechnology, Biology Faculty of Hubei University, Hubei University, Wuhan 430062, Chinawangfei@hubu.edu.cn (F.W.);; 2 Hubei Biopesticide Engineering Research Center, Hubei Academy of Agricultural Sciences, Biopesticide Branch of Hubei Innovation Centre of Agricultural Science and Technology, Wuhan 430064, China

**Keywords:** *Taq* DNA polymerase, *CL7*, fusion DNA polymerase, PCR amplification

## Abstract

DNA polymerases are important enzymes that synthesize DNA molecules and therefore are critical to various scientific fields as essential components of in vitro DNA synthesis reactions, including PCR. Modern diagnostics, molecular biology, and genetic engineering require DNA polymerases with improved performance. This study aimed to obtain and characterize a new *CL7*-*Taq* fusion DNA polymerase, in which the DNA coding sequence of *Taq* DNA polymerase was fused with that of *CL7*, a variant of *CE7* (Colicin E7 DNase) from *Escherichia coli*. The resulting novel recombinant open reading frame was cloned and expressed in *E. coli*. The recombinant *CL7*-*Taq* protein exhibited excellent thermostability, extension rate, sensitivity, and resistance to PCR inhibitors. Our results showed that the sensitivity of *CL7*-*Taq* DNA polymerase was 100-fold higher than that of wild-type *Taq*, which required a template concentration of at least 1.8 × 10^5^ nM. Moreover, the extension rate of *CL7*-*Taq* was 4 kb/min, which remarkably exceeded the rate of *Taq* DNA polymerase (2 kb/min). Furthermore, the *CL7* fusion protein showed increased resistance to inhibitors of DNA amplification, including lactoferrin, heparin, and blood. Single-cope human genomic targets were readily available from whole blood, and pretreatment to purify the template DNA was not required. Thus, this is a novel enzyme that improved the properties of *Taq* DNA polymerase, and thus may have wide application in molecular biology and diagnostics.

## 1. Introduction

Discovered in the 1980s, polymerase chain reaction (PCR) has become one of the most significant technological advancements in the fields of biology and clinical medicine. Technology that detects a broad range of bacteria or viruses based on PCR is widely applied for the screening of clinical, environmental, and manufacturing samples to diagnose infectious diseases or to detect bacterial contamination. Therefore, PCR technology plays a vital role in molecular biology, genetic engineering, and diagnostics [1]. The *Taq* DNA polymerase that is now widely used in PCR was first isolated from the extreme thermophilic bacteria *Thermus aquaticus* YT-1 [2].

Currently, the *Taq* DNA polymerase is crucial for almost all PCR-based techniques, including RT-PCR and digital PCR. However, the *Taq* DNA polymerase has the drawback of a relatively higher error frequency than other enzymes [3]. Usually, product yields will decrease when the amplification fragment becomes longer than 1 kb due to the relatively low processivity and thermostability of the wild-type enzyme. Generally, the efficiency of amplification by the *Taq* polymerase for targets shorter than 1 kb is approximately 80% [4]. However, *Taq* DNA polymerases become completely inhibited when the PCR mixture contains 0.2% blood. It seems that hemoglobin and lactoferrin have important roles in inhibiting the amplification process [5]. Therefore, these drawbacks limit the applications of *Taq* DNA polymerase.

Recently, a novel strategy was employed to overcome these limitations [6]. Generating fusion proteins with *Taq* DNA polymerases and a thermostable DNA-binding protein such as the Sso7d DNA-binding protein from *Sulfolobus solfataricus* has been shown to enhance the processivity [7], thermostability, and overall stability compared with the wild-type *Taq* polymerase. The purified S-*Taq* protein has shown acceptable limits of host genomic DNA levels without the use of DNases or other DNA precipitating agents, which highlights its potential for use in PCR-based diagnostics, in situ PCR, and forensic science [7].

A previous study also found that adding 0.6% bovine serum albumin (BSA) to reaction mixtures containing the *Taq* DNA polymerase reduced the inhibitory effect of blood and allowed for DNA amplification in the presence of 2% instead of 0.2% blood [8]. Furthermore, BSA was found to be the most efficient amplification facilitator. As rapid and simple diagnostic methods are urgently required for blood analyses, modifications to *Taq* DNA polymerase that enhance its tolerance to inhibition by blood are significant to medical diagnostics. Mutations to the *Taq* DNA polymerase that render it resistant to inhibition by blood components are now commercially available [9]. Additionally, there are *Taq* polymerase mutants that are used in PCR-based tests of blood and soil samples that are widely used for diagnostics and forensic analyses and do not require pretreatment to purify the template DNA, and there are others that allow increased dye concentration to overcome fluorescence background and quenching in real-time PCR analyses of blood. Because of deficiencies in the 3′-5′ exonuclease domain, the *Taq* DNA polymerase is widely applied to ARMS-PCR to detect single nucleotide polymorphisms (SNPs).

In recently reported studies, the *Taq* polymerase was fused to DNA binding proteins to improve other properties [10]. In this paper, we tried to apply the variant of *CE7* (Colicin E7 DNase) to achieve that effect. The *CE7* protein belongs to the category of highly toxic proteins because of its DNase domains (16 kDa). The wild *CE7* could bind with DNA and degrade it. Considering that the DNase activity of *CE7* is lethal to the cells, constructing the inactive variants of *CE7* is an appropriate option. Here, the active site histidine residues and DNA-binding residues were mutated to produce the *CL7* variant lacking the catalytic activities. That paper said that the *CL7* variant also lacked DNA-binding ability. However, we found that *CL7* could increase the stability of *Taq*. By taking the features of *CL7*, we fused *CL7* with *Taq* to improve the properties of the *Taq* polymerase. We demonstrated and functionally characterized the fusion enzymes and demonstrated that they showed improved processivity, sensitivity, amplification rates, and eliminated pre-PCR treatment steps. Finally, we demonstrated the practical benefits of PCR applications for enhancing the sensitivity of the polymerase.

## 2. Materials and Methods

### 2.1. Construction of Recombinant Plasmids

First, DNA coding sequences of the DNA polymerase from Thermus aquaticus (GenBank: P19821.1) and of *CL7*, which is a mutant of *CE*7 from *E. coli* (GenBank: CP018986.1), were obtained [11]. The two fragments were synthesized by GeneCreate (Wuhan, China). The designed primers for amplification are listed in Table 1. The open reading frame synthesis procedure was performed as described previously. PCR amplification was then used to obtain two products: the *Taq* DNA polymerase open reading frame (2493 bp) and the *CL7* gene (390 bp). The pET-30 vector was digested with the ndonucleasee Xba I (Takara, Shiga, Japan), and the product was purified using the DNA Gel Extraction Kit (Promega, Madison, WI, USA). Then, the PCR products were mixed together with the digested pET-30 vector. Thus, the open reading frame encoding a fragment of *Taq* DNA polymerase was cloned into the pET-30 vector to generate a pET30/*Taq* plasmid. This led to the expression of a *Taq* DNA polymerase fusion protein with a C-terminal 6× histidine tag. Finally, T5 cloning (New England Biolabs, Ipswich, MA, USA) was performed, in which *CL7* was fused to the N-terminus of *Taq* DNA polymerase with the 7-amino acid linker (ENLYFQG) and a 6× His tag to the C-terminus, resulting in plasmid pET30/*CL7*-*Taq*. This was necessary for the purification of a recombinant protein by metal affinity chromatography. Nucleotide sequences of the resulting recombinant plasmids, pET30/*Taq* and pET30/*CL7-Taq*, were confirmed by DNA sequencing (Sangon, Shanghai, China).

### 2.2. Protein Expression and Purification

Plasmids encoding *Taq* and *CL7-Taq* were used to transform the *E. coli* BL21 strain (DE3). Luria–Bertani (LB) medium was prepared. *E. coli* containing recombinant plasmid were grown to an A600 of 0.6 in LB containing 50 μg/mL kanamycin at 37 °C. Then, isopropyl-β-D-1-thiogalactopyranoside (IPTG) was added at a final concentration of 1 mg/mL to induce *Taq* or *CL7*-*Taq* expression from the T7 promoter with shaking at 18 °C. After 12 h of incubation, the cells were centrifuged at 7000× *g* for 5 min, and the pellets were resuspended in 20 mL of lysis buffer (20 mM Tris-HCl [pH 9.0], 0.5 M NaCl, and 10 mM imidazole). Protein complexes were extracted by ultrasonic decomposition and the insoluble debris was removed by centrifugation at 12,000× *g* and 4 °C for 20 min.

For heat treatment, the cleared lysate was immersed in a 75 °C orbital water shaker for 30 min, cooled on ice for 20 min, and then, the denatured host proteins were removed by centrifugation at 12,000× *g* and 4 °C for 20 min. Following heat treatment, the exogenous proteins were purified in a one-step process using the Ni^2+^-affinity chromatographic technique. A His-bind resin and His-bind buffer kit (GE Life Sciences, Chicago, IL, USA) were used to purify the His-tagged proteins according to the manufacturer’s instructions. The supernatant and produced enzyme were put into a column containing Ni–nitrilotriacetic acid (Ni-NTA) agarose (GE Life Sciences), which was previously prepared and equilibrated with lysis buffer. After binding to the Ni-NTA agarose column, the recombinant proteins were washed twice with washing buffer (20 mM Tris-HCl [pH 9.0], 0.5 M NaCl, and 50 mM imidazole), and then eluted with elution buffer (20 mM Tris-HCl [pH 9.0], 0.5 M NaCl, and 200 mM imidazole). An equal volume of each buffer (20 μL) was loaded onto a 12% (*w*/*v*) polyacrylamide gel for SDS-PAGE [12], followed by staining with Coomassie Brilliant Blue G-250. Finally, the eluted fractions were dialyzed three times with storage buffer (20 mM Tris-HCl [pH 8.0], 100 mM KCl, and 0.2 mM EDTA). Protein concentrations were measured using the Bradford method.

### 2.3. DNA Polymerase Activity Assay

The DNA polymerase activity of purified proteins was assayed using the EvaEZ Fluorometric Polymerase Activity Assay Kit (Biotium, Hayward, CA, USA). All assays were conducted in an isothermal reaction at 60 °C using a CFX Real-Time PCR instrument (Bio-Rad, Hercules, CA, USA) in accordance with the definition of one unit of enzyme activity (“One unit of DNA polymerase activity is usually defined as the amount of enzyme that will produce 10 nmol of nucleotides during a 30-min incubation”). Enzymatic activity was determined relative to a commercial *Taq* DNA polymerase (Thermo Fisher Scientific, Waltham, MA, USA) with an activity of 1 U/μL. When the DNA polymerase was active, the primer was extended to form a double-stranded product that bound the EvaGreen dye, resulting in increased fluorescence. The rate of increase is positively correlated with polymerase activity [13].

### 2.4. Optimization of PCR Amplification

To optimize the amplification process, polymerase activity was measured using various concentrations of MgCl_2_, KCl, and (NH_4_)_2_SO_4_ in the buffer as well as various pHs. All PCR reactions were performed using 0.2 mM of each dNTP and 0.4 mM of each primer and of the pET30a-GFP plasmid DNA as template, containing a known target sequence (PCR product of 445 bp). PCR assays were performed using 1 U of purified *CL7*-*Taq* or *Taq* DNA polymerase in a 20 μL reaction mixture containing 1 ng of DNA template. The PCR conditions were as follows: 3 min at 95 °C, and then 25 cycles of 15 s at 95 °C, 15 s at 60 °C, and 30 s at 72 °C. To determine the optimum MgCl_2_ concentration, PCR was performed at various pHs (7.0–9.0) with the use of the Tris–HCl buffer. Then, we used the Tris-HCl buffer (pH 8.0) containing increasing concentrations of MgCl_2_ (0–9 mM) to detect the enzyme activity. Furthermore, PCR was performed with various concentrations of KCl (10–90 mM) and (NH_4_)_2_SO_4_ (10–90 mM).

Temperature stability was also assayed [14]. One unit of purified *CL7*-*Taq* and *Taq* DNA polymerases was heated at 99 °C for 10, 20, 30, 40, 50, and 60 min, and at 95 °C for 1, 2, 3, 4, and 5 h. Then, the same amount of enzyme was used to amplify a 445 bp target fragment under the optimal reaction buffer (in the same PCR conditions determined in the optimization process): 20 mM Tris-HCl (pH 8.0), 4 mM MgCl_2_, 10 mM (NH_4_)_2_SO_4_, and 20 mM KCl.

### 2.5. Measuring the PCR Amplification Rate

PCR amplification rates were measured using the protocol described by [15]. *CL7*-*Taq* and *Taq* DNA polymerases were used to amplify PCR products of 1, 2, 3, and 4 kb under the conditions determined in the optimization process and using pET23a/dcas9 plasmid DNA as a template, which was recombined in our laboratory. PCR amplification started with an initial denaturation at 95 °C for 3 min and included 25 cycles of 30 s at 95 °C, 30 s at 60 °C, and 60 s at 72 °C.

### 2.6. Sensitivity

Following sufficient modifications, DNA polymerase sensitivity (affinity for template) was measured using the protocol by [16]. PCR was conducted under conditions optimized for the fusion polymerases *CL7*-*Taq* and *Taq*. We used pET23a-GFP plasmid as a template along with the primers 5′-TGGTCTTCAATGCTTTGCGAGATAA-3′ (forward) and 5′-CTTTTCGTTGGGATCTTTCG-3′ (reverse). The product of the reaction was 445 bp. The reaction was conducted at decreasing template concentrations (serial 10-fold dilutions of the template) and included an initial denaturation at 95 °C for 3 min followed by 35 cycles of 30 s at 95 °C, 30 s at 60 °C, and 90 s at 72 °C. The amplified fragments were analyzed in a 1.5% agarose gel stained with ethidium bromide.

### 2.7. Resistance to Inhibitors

The effect of PCR inhibitors such as heparin (Sigma-Aldrich, St. Louis, MO, USA) at a range from 16 to 1 μg, lactoferrin (Sigma-Aldrich) at a range from 4 to 0.5 μg, and blood at a range from 8% to 1% on the catalytic activity of the *CL7*-*Taq* and *Taq* DNA polymerases was assessed by a PCR reaction using human genomic DNA as a template and the specific β-actin primers 5′-AGAGATGGCCACGGCTGCTT-3′ (forward) and 5′-ATTTGCGGTGGACGATGGAG-3′ (reverse) [16]. The amplified fragments were analyzed in a 1.5% agarose gel stained with ethidium bromide.

### 2.8. Fidelity of CL7-Taq

A PCR-based forward mutation assay was performed by Wang Fei [15]. The mannanase activity was applied to measure the error rate of the *CL7*-*Taq* DNA polymerase. PCR was carried out by *CL7*-*Taq* and *Taq* polymerases under optimized conditions. The resulting PCR products were treated with *Dpn*I, subjected to agarose gel DNA purification, and transformed into *E. coli* XL10-Gold. These clones were transferred to LB plates containing 100 mM arabinose and 0.03% (*m*/*v*) trypan blue (which gives the plates a blue appearance) and 0.5% (*m*/*v*) Konjac Flour as the substrate for the mannanase. A clear zone was visible around mannanase-expressing colonies capable of hydrolyzing the Konjac glucomannan. A single colony of *E. coli* XL10-Gold that contained the plasmid pBAD-man was used as a positive control, and a single colony of *E. coli* XL10-Gold that contained the plasmid pBAD-His-GFP was used as a negative control. Hydrolysis holes with different sizes were observed and measured after 12 h of culture. Error rates were calculated based on the sizes of the hydrolysis holes. Clones that produced much larger or smaller hydrolysis holes were considered mutants.

### 2.9. PCR Amplification under Standard Buffer Conditions

*CL7*-*Taq* and *Taq* DNA polymerases (Takara, Shiga, Japan) were used to amplify PCR products of 1, 2, 3, and 4 kb under standard buffer conditions and using pET23a/dcas9 plasmid DNA as a template. The reactions performed here used the provided *Taq* enzyme buffer (Takara, Shiga, Japan). The optimum condition was obtained as above: 20 mM Tris-HCl (pH 8.0), 4 mM MgCl_2_, 10 mM (NH_4_)_2_SO_4_, and 20 mM KCl.

## 3. Results and Discussion

### 3.1. Expression and Purification of Taq and CL7-Taq

**Figure 1:**
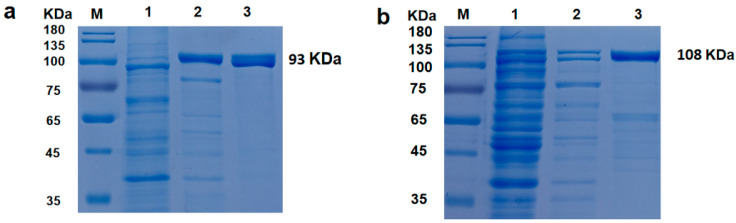
Expression and purification of the *Taq* (**a**) and the *CL7-Taq* (**b**) polymerases. The proteins were analyzed on a 12% polyacrylamide gel (SDS-PAGE). Lane M, pageruler (Thermo Scientific, Waltham MA, USA), with the molecular mass of proteins marked. Lane 1, sonicated extract of induced cells; lane 2, heat treatment; lane 3, purified protein after elution with storage buffer.

The recombinant strains were cultured and induced. After induction, the cells were harvested and sonicated. The recombinant DNA polymerases were then purified by passing the heat-denatured supernatant through a His-Bind Ni^2+^ affinity column. After each purification step, the purity of the DNA polymerase was monitored by SDS-PAGE (Figure 1), which separated the following major protein bands: 93 and 108 kDa for *Taq* and *CL7*-*Taq*, respectively; this result was in agreement with the molecular masses of 92.7 and 107.7 kDa that were calculated based on the amino acid sequences. The *E.coli* overexpression system used in this study enabled the production of 720 mg of the *Taq* polymerase and 620 mg of the *CL7*-*Taq* fusion protein per 1 L of induced culture. After being measured by the EvaEZ Fluorometric Polymerase Activity Assay Kit, the specific activities of the purified *Taq* and *CL7*-*Taq* DNA polymerases were found to be 1426.4 ± 18 and 1572.6 ± 21 U/mg, respectively. These results indicate that the *CL7* fusion had a positive effect on the relative activity of the *Taq* DNA polymerase. The production efficiency of *Taq* and *CL7*-*Taq* DNA polymerases in this study was satisfactory.

### 3.2. Characterization of Taq and CL7-Taq

**Figure 2:**
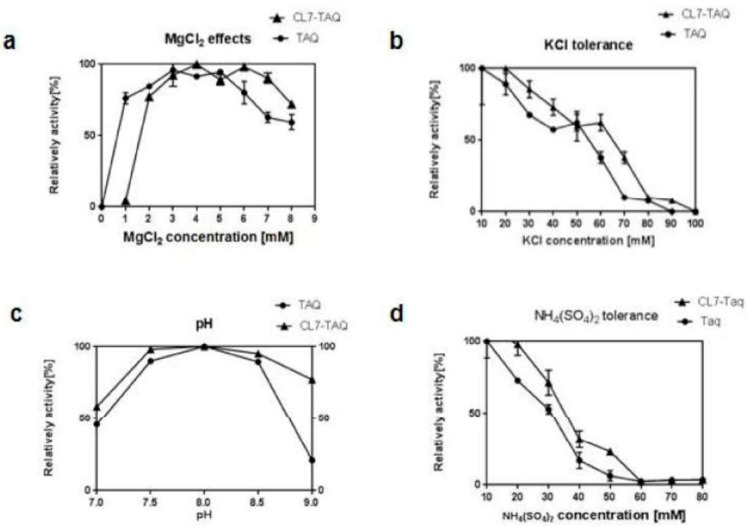
Characterization of the fusion *Cl7-Taq* DNA polymerase in comparison to the *Taq* DNA polymerase. The effect of (**a**) MgCl_2_, (**b**) KCl, (**c**) pH, and (**d**) (NH4)_2_SO_4_. Error bars for *Cl7-Taq* DNA polymerase have the end bar; for *Taq* DNA polymerase, error bars do not have the end bar.

For characterization purposes, the activity of the *Taq* and *CL7*-*Taq* DNA polymerases was measured by PCR using various buffer compositions and concentrations of MgCl_2_, KCl, and (NH_4_)_2_SO_4_ and various pHs (Figure 2). With a commonly used alpha level of 0.05, we would reject the null hypothesis in both cases, meaning there is statistically significant evidence that pH affects enzyme activity. The specific *p*-value tells us the strength of this evidence.

The effect of pH on the activity of *Taq* and *CL7*-*Taq* DNA polymerases was evaluated using Tris-HCl buffers of pH ranging from 7.0 to 9.0. Both polymerases had the highest enzyme activity at pH 8.0 (Figure 2c). The activity of *Taq* and *CL7*-*Taq* DNA polymerases was closely related to the concentration of MgCl_2_, which was optimal between 1 and 8 mM and from 2 to 8 mM, respectively (Figure 2a). DNA polymerase activity was completely inhibited when KCl concentrations surpassed 70 mM for *Taq* and 80 mM for *CL7*-*Taq* (Figure 2b). The activity of *Taq* and *CL7*-*Taq* DNA polymerases was also strongly affected by (NH_4_)_2_SO_4_ and was completely inhibited at concentrations over 30 and 40 mM, respectively (Figure 2d). The fusion of *CL7* to the *Taq* DNA polymerase resulted in higher tolerance of the enzyme to salt inhibition. All of these findings were statistically significant with *p*-values below 0.05. We also report F-statistics and degrees of freedom from the ANOVA to prove the analysis.

Based on these results, we found that the optimal PCR buffer for the *CL7*-*Taq* DNA polymerase consisted of 20 mM Tris-HCl (pH 8.0), 4 mM MgCl_2_, 10 mM (NH_4_)_2_SO_4_, and 20 mM KCl.

### 3.3. PCR Amplification Rate and Processivity

**Figure 3:**
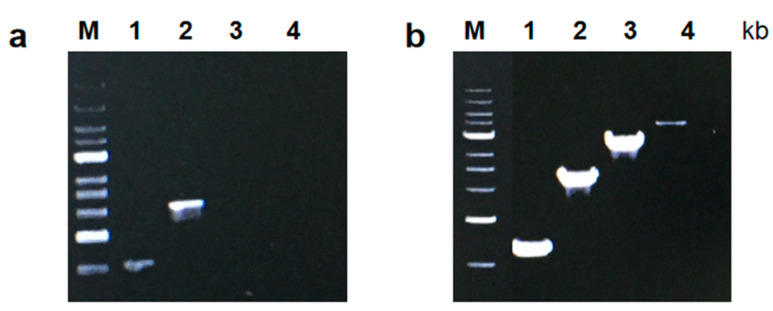
Comparison of PCR amplification rates of *Taq* DNA polymerase (**a**) and the fusion *Cl7*-*Taq* DNA polymerase (**b**). The PCR products’ size are shown at the top. Lane M, GeneRuler 1 kb DNA Ladder marker (250–10,000 bp) (Thermo Scientific, London, UK). The amplified products were analyzed in a 0.8% agarose gel stained with ethidium bromide.

To measure the PCR amplification rate, we designed tests similar to those previously published [15]. The results showed that the fusion *CL7*-*Taq* DNA polymerase replicated template strands at a faster rate than the *Taq* DNA polymerase (Figure 3). The data showed that *CL7*-*Taq* extended a 4000 bp product in 60 s, while the *Taq* DNA polymerase required 1 min for a 2000 bp product. This showed that the fusion of the *CL7* protein with *Taq* DNA polymerase was twofold more efficient than *Taq* DNA polymerase without *CL7*, meaning DNA amplification required less time.

### 3.4. Sensitivity

**Figure 4:**
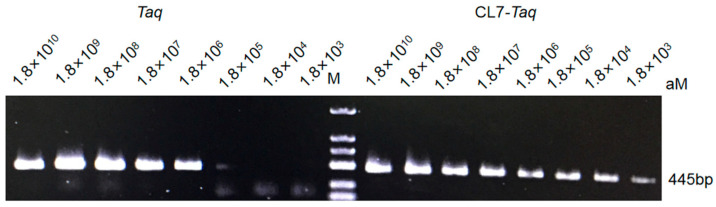
Amplification sensitivity of *Taq* and *CL7 Taq*. Electrophoretic separation showing the products of plasmid DNA amplification as a function of a template concentration with the use of the fusion polymerase *CL7-Taq* polymerase and the *Taq* polymerase. Lane M, DL2000 (100–2000 bp) (TsingKe, Beijing, China). The amplified products were analyzed in a 1% agarose gel stained with ethidium bromide.

To monitor enzyme sensitivity, we used the protocol published previously in [15]. Sensitivity was measured by PCR with *CL7*-*Taq* and *Taq* polymerases and 10-fold serial dilutions of the template; the product size was 445 bp. The results showed that the fusion polymerase was more sensitive than the wild-type polymerase. In the case of the *CL7*-*Taq* DNA polymerase, it was sufficient to use 1.8 × 10^3^ nM of plasmid DNA, while the *Taq* polymerase required at least 1.8 × 10^5^ nM of plasmid. These data showed that the sensitivity of the *CL7*-*Taq* DNA polymerase was improved 100-fold compared with the *Taq* polymerase (Figure 4).

### 3.5. Thermostability of the DNA Polymerases

**Figure 5:**
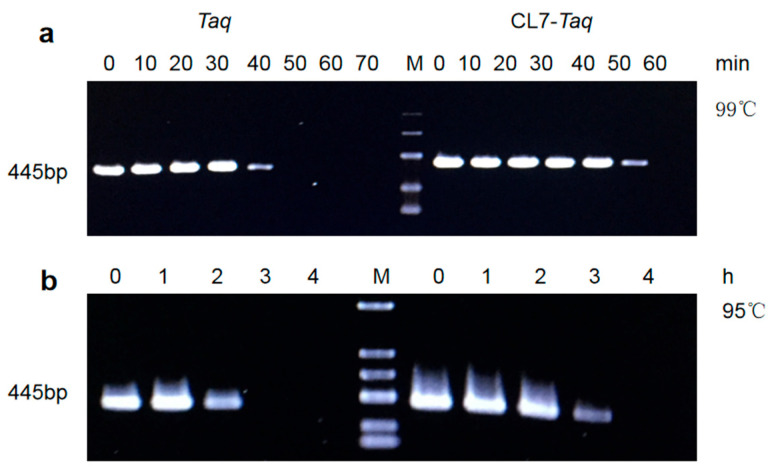
Thermoresistant properties of *Taq* and CL2 *Taq*. Residual activity was assayed by PCR after incubation at 99 °C (**a**) or 95 °C (**b**). Differences in the amplification efficiency for the fusion *Cl7*-*Taq* DNA polymerase and *Taq* DNA polymerase after incubation at 99 °C for 0–70 min and incubation at 95 °C for 0–4 h, respectively. Lane M: the DNA molecular size marker DL2000 (TsingKe, China).

To determine the thermostability of *Taq* and *CL7*-*Taq* DNA polymerases, the enzymes were monitored for decreased activity after preincubation at 95 °C and 99 °C. These experiments revealed that the *CL7*-*Taq* DNA polymerase had remarkably higher thermostability. Our data showed that *Taq* and *CL7*-*Taq* DNA polymerases were functional after 30 and 50 min at 99 °C, respectively (Figure 5a). Additionally, *Taq* and *CL7*-*Taq* DNA polymerases remained active after 2 and 3 h at 95 °C, respectively (Figure 5b).

### 3.6. Tolerance of the DNA Polymerases to PCR Inhibitors

**Figure 6:**
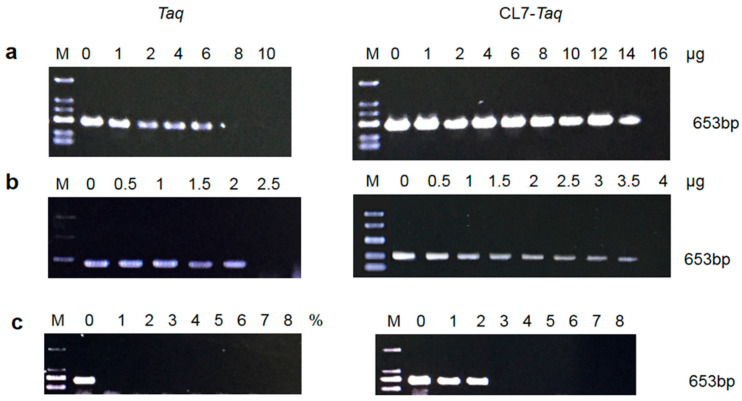
The effect of heparin (**a**), lactoferrin (**b**), and blood (**c**) on DNA amplification using human genomic DNA as a template and primers for β-actin. Control reactions were performed without any inhibitors. Lane M, DL2000 (100–2000 bp) (TsingKe, China). The amplified products were analyzed in a 1% agarose gel stained with ethidium bromide.

To determine the limiting concentration of PCR inhibitors, the fusion *CL7*-*Taq* and the *Taq* DNA polymerases were PCR-tested for their resistance to serial dilutions of blood, lactoferrin, and heparin, which have been reported to be PCR inhibitors in many publications [9]. The results showed that the *CL7*-*Taq* and *Taq* polymerases remained active in the presence of 3.5 μg and 2 μg lactoferrin, respectively (Figure 6b), and 14 μg and 6 μg of heparin, respectively (Figure 6a). *CL7*-*Taq* and *Taq* were resistant to 2% and 0% blood, respectively (Figure 6c). Together, these findings revealed that the fusion *CL7*-*Taq* DNA polymerase was significantly more resistant to blood, lactoferrin, and heparin compared with the *Taq* enzyme.

### 3.7. Fidelity of CL7-Taq

The results showed that *CL7-Taq* had an error rate of 1.28%, which was similar to the error rates (1.29%) of commercial *Taq* polymerase. It means that these two enzymes had the same fidelity. The fusion protein *CL7* could not improve the fidelity of *Taq* polymerase.

### 3.8. PCR Amplification Using Standard Buffer

**Figure 7:**
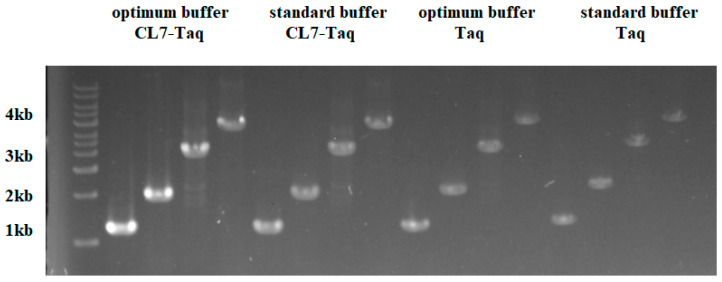
Comparison of PCR amplification of the fusion *CL7-Taq* DNA polymerase and the *Taq* DNA polymerase using optimum buffer or standard buffer.

Obviously, the results (Figure 7) reveal that *CL7-Taq* showed the highest activity under the optimum conditions. It even performs better than *Taq* polymerase in standard buffer. So *CL7-Taq* can replace *Taq* in many scenarios and can be commercialized.

## 4. Discussion

PCR technology has been widely applied in the fields of molecular biology, genetic engineering, and diagnostics [8]. PCR amplification efficiency is strongly dependent on the properties of the DNA polymerase and reaction conditions. Wild-type DNA polymerase has some drawbacks; therefore, modern diagnostic methods and genetic engineering techniques require modified DNA polymerases with better properties, such as those that possess higher sensitivity and/or amplification rates. For this reason, we engineered fusions of the DNA polymerase to enhance its ability. In this study, the N-terminus of the *Taq* DNA polymerase was fused with the thermoduric *CL7* protein using a seven-amino-acid linker (Gly-Asn-Leu-Tyr-Phe-Gln-Cys).

The most common challenges during the amplification of environmental and blood samples are inhibitors present in the tested material [17]. Our observations of successful amplification of the *β-actin* open reading frame directly from human blood using *CL7*-*Taq* is very promising and opens up a new avenue for this polymerase as a valuable tool for medical diagnostics and forensic science, where sample availability is minimal. We found that the enhanced salt tolerance of *CL7*-*Taq* is responsible for its successful use in direct PCR of the human genome without preprocessing. Previous studies have shown that PCR inhibitors alter DNA or block enzymes though these pathways, such as inhibiting the active site or blocking access to the active site for cofactors such as Mg^2+^ ions [8,18]. Therefore, inhibitors either weaken the efficiency of PCR amplification or block it completely. However, commercially available native enzymes are not always able to deal with these PCR issues. Our data showed that the fusion *CL7*-*Taq* DNA polymerase exhibited a higher tolerance to PCR inhibitors (blood, lactoferrin, and heparin) compared with the *Taq* DNA polymerase. Therefore, the *Taq* DNA polymerase containing *CL7* had better performance in the amplification of complicated templates.

Our data showed that the fusion of *CL7* to the *Taq* DNA polymerase improved enzymatic properties such as thermostability, amplification rate, and template sensitivity. In thermostable tests, we found that the thermostability of the *CL7*-*Taq* DNA polymerase was remarkably higher than that of the *Taq* DNA polymerase. The thermostability of *CL7*-*Taq* was over 1 h longer than that of the *Taq* DNA polymerase at 95 °C. Mg^2+^ and other salts were critical components of PCR reactions; thus, optimizing their concentrations was essential for native DNA polymerase. Unlike the *Taq* DNA polymerase, the *CL7*-*Taq* DNA polymerase exhibited sufficient amplification efficiency within a wide range of Mg^2+^ concentrations. Furthermore, the *CL7*-*Taq* DNA polymerase had a satisfactory amplification efficiency at various concentrations of KCl and (NH_4_)_2_SO_4_. Thus, the salt tolerance of *CL7*-*Taq* was remarkably enhanced.

Our observation that *CL7-Taq* had higher sensitivity than *Taq* makes it an attractive enzyme for PCR-based diagnostics. Therefore, it is tempting to speculate that one could explore using *CL7-Taq* for ARMS-PCR to detect SNPs or common DNA viruses such as HPV, Herpesvirus, and Parvoviruses.

## 5. Conclusions

In this study, we aimed to obtain and characterize a new *CL7-Taq* fusion DNA polymerase, in which the DNA coding sequence of the *Taq* DNA polymerase was fused with that of *CL7*, a variant of *CE7* (Colicin E7 DNase) from *Escherichia coli*. Our results showed that the sensitivity of the *CL7-Taq* DNA polymerase was 100-fold higher than that of the wild-type *Taq*, which required a template concentration of at least 1.8 × 10^5^ nM. Moreover, the extension rate of *CL7-Taq* was 4 kb/min, which remarkably exceeded the rate of the *Taq* DNA polymerase (2 kb/min). The recombinant *CL7-Taq* protein exhibited excellent thermostability, extension rate, sensitivity, and resistance to PCR inhibitors. Thus, this is a novel enzyme that improved the properties of the *Taq* DNA polymerase and thus may have wide application in molecular biology and diagnostics.

## Figures and Tables

**Table 1:** Comparison of the fidelities of *CL7-Taq* and *Taq.*

Enzymes	No. of Clear Zones		Mutation Frequency (%)
	Mutant	Total	
*CL7-Taq*	154	12,000	1.28
*Taq*	162	12,500	1.29

## Data Availability

The original contributions presented in the study are included in the article, further inquiries can be directed to the corresponding authors.

## Abbreviations

*CL7*: a mutant of the *CE7* protein; PCR, polymerase chain reaction.
